# Long-term interdisciplinary therapy reduces endotoxin level and insulin resistance in obese adolescents

**DOI:** 10.1186/1475-2891-11-74

**Published:** 2012-09-18

**Authors:** Fábio S Lira, Jose C Rosa, Gustavo D Pimentel, Ronaldo V Santos, June Carnier, Priscila L Sanches, Aline de Piano, Claudio T de Souza, Lian Tock, Sergio Tufik, Marco T de Mello, Marília Seelaender, Claudia M Oller do Nascimento, Lila M Oyama, Ana R Dâmaso

**Affiliations:** 1 Departamento de Fisiologia, Rua Botucatu nÂº 862 Vila Clementino, São Paulo, Brazil; 2 Departamento de Biociências, Rua Marselhesa n Vila Clementino, São Paulo, Brazil; 3 Departamento de Psicobiologia, Rua Marselhesa n Vila Clementino, São Paulo, Brazil; 4 Da Universidade Federal de São Paulo–UNIFESP, São Paulo. Grupo de Metabolismo e Câncer, ICB-Universidade de São Paulo–USP, São Paulo, Brazil; 5 Laboratory of Exercise Biochemistry and Physiology, Health Sciences Unit, University of Southern Santa Catarina, Criciúma, SC, Brazil

**Keywords:** Obesity, Cytokines, Endotoxin, Insulin resistance, Interdisciplinary therapy

## Abstract

**Aim:**

The purpose of the present study was to assess the dietary fat intake, glucose, insulin, Homeostasis model assessment for insulin resistance HOMA-IR, and endotoxin levels and correlate them with adipokine serum concentrations in obese adolescents who had been admitted to long-term interdisciplinary weight-loss therapy.

**Design:**

The present study was a longitudinal clinical intervention of interdisciplinary therapy. Adolescents (n = 18, aged 15–19 y) with a body mass index > 95th percentile were admitted and evaluated at baseline and again after 1 year of interdisciplinary therapy. We collected blood samples, and IL-6, adiponectin, and endotoxin concentrations were measured by ELISA. Food intake was measured using 3-day diet records. In addition, we assessed glucose and insulin levels as well as the homeostasis model assessment for insulin resistance (HOMA-IR).

**Results:**

The most important finding from the present investigation was that the long-term interdisciplinary lifestyle therapy decreased dietary fat intake and endotoxin levels and improved HOMA-IR. We observed positive correlations between dietary fat intake and endotoxin levels, insulin levels, and the HOMA-IR. In addition, endotoxin levels showed positive correlations with IL-6 levels, insulin levels and the HOMA-IR. Interestingly, we observed a negative correlation between serum adiponectin and both dietary fat intake and endotoxin levels.

**Conclusions:**

The present results indicate an association between dietary fat intake and endotoxin level, which was highly correlated with a decreased pro-inflammatory state and an improvement in HOMA-IR. In addition, this benefits effect may be associated with an increased adiponectin level, which suggests that the interdisciplinary therapy was effective in improving inflammatory pathways.

## Introduction

Saturated and trans fatty acids intake leads to inflammation, insulin resistance and a gain in body mass. Systemic low-level inflammation has been suggested to be both a cause and a consequence of comorbidities associated with obesity [1,2]. Recently, studies have proposed that the microbial ecology in humans could be an important factor in determining energy homeostasis (i.e., obesity, diabetes, and fatty liver) [3-9]. Lipopolysaccharide (LPS), which is also referred to as endotoxin, has been implicated as a potent inducer of inflammation, and LPS increases tumor necrosis factor alpha (TNF-α) and interleukin 6 (IL-6) and reduces adiponectin levels. In normal circumstances, only small amounts of endotoxin cross from the intestinal lumen into systemic circulation, and the absorbed endotoxin is rapidly removed by monocytes, particularly resident Kupffer cells within the liver. Emerging evidence has indicated that chronic elevation of serum endotoxin levels may play a role in insulin-resistant states and obesity [4,5,10].

Interestingly, some authors described how physical inactivity leads to the accumulation of visceral fat and the activation of a network of inflammatory pathways that promote the development of insulin resistance, atherosclerosis, obesity, neurodegeneration, and tumor growth (i.e., the development of diseases belonging to the “diseasome of physical inactivity”) [11,12].

Recently, our group has shown that long-term interdisciplinary lifestyle therapy is effective in controlling the psychological and physiological alterations that are commonly observed in obese patients [13-21]. Despite promising results, few studies have addressed the effects of long-term interdisciplinary intervention on dietary fat intake and endotoxin levels and their correlation with insulin resistance and adipokine levels.

## Materials and methods

### Population

Fifty-four adolescents were invited to participate in a 1-year-long Interdisciplinary Obesity Program at the Federal University of São Paulo- Paulista Medical School to promote changes in their sedentary lifestyle and nutritional habits. The basic requirements for participation were motivation and high attendance at the therapy sessions. Thirty-nine adolescents participated until the end of the therapy. The main reasons for dropping out in our study were financial and family problems, followed by school and job opportunities. For the present study, we used as exclusion criteria the lost fat mass < 5% at the end of therapy, where we selected 18 obese adolescents who lost more than 5% fat mass (the range was 5.4% to 22.5% fat mass).

Selected 18 obese adolescents were evaluated at baseline and after long-term (1 year) weight loss intervention. The present study was conducted in accordance with the principles of the Declaration of Helsinki and was formally approved by the Institutional Ethical Committee (#0135/04). Informed consent was obtained from all subjects and/or their parents, and the agreement of the adolescents and their families to participate was voluntary.

The ages of the 18 participants ranged from 15–19 years (16.6 ± 1.67 years), and the average body mass index (BMI) was 37.98 ± 4.60 kg/m^2^ (7 boys and 11 girls). All participants met the inclusion criteria of postpubertal Stage V, based on the Tanner stages [22], and of obesity (BMI > 95th percentile) according to the Centers for Disease Control and Prevention (CDC) reference growth charts. Noninclusion criteria included identifiable genetic, metabolic or endocrine disease or previous drug utilization [23].

### Study protocol and medical screening

Subjects were medically screened, and we assessed their pubertal stage and recorded their anthropometric measures (i.e., height, weight, BMI and body composition). The endocrinologist completed a clinical interview, which included questions to determine eligibility based on inclusion and exclusion criteria. Blood samples were collected and analyzed, and an ultrasound (US) was performed. The procedures were scheduled for the same time of day for all subjects to remove any influence of diurnal variations. After the initial screening, obese adolescents started the interdisciplinary weight loss program (described in a later section).

### Anthropometric measurements and body composition

Subjects were weighed on a Filizola scale while wearing light clothing and no shoes, and their weight was recorded to the nearest 0.1 kg. Height was measured using a wall-mounted stadiometer (Sanny, model ES 2030) to the nearest 0.5 cm. Body mass index was calculated as body weight (wt) divided by height (h) squared (wt/ht^2^). Body composition was estimated by plethysmography using the BOD POD body composition system (version 1.69, Life Measurement Instruments, Concord, CA), which is the most advanced technique available for assessing body composition. The patented air displacement plethysmography used by the BOD POD and PEA POD is similar in principle to hydrostatic (or "underwater") weighing. The obvious difference between them is that air is more convenient and comfortable than water, and air displacement plethysmography provides a much simpler and safer testing environment, better reliability and significantly improved repeatability and accuracy [24].

### Serum analysis

Blood samples were collected in the outpatient clinic at approximately 0800 h after an overnight fast. Adipokine (IL-6 and adiponectin) concentrations were measured using commercially available ELISA kits from eBioscience, Inc. (San Diego, CA, USA) and R&D Systems (USA) according to the manufacturer’s manuals.

Fasting insulin concentrations were determined using commercially available ELISA kits from Millipore (Millipore Corporate Headquarters: 290 Concord Road, Billerica, MA 01821), and glucose concentrations were determined by an enzymatic method (Labtest®). Homeostasis model assessment for insulin resistance (HOMA-IR) was calculated with assessed values of glucose and insulin.

### Measurement of circulating endotoxin levels

Plasma endotoxin was assayed using a chromogenic limulus amebocyte lysate (LAL) test, which is a quantitative test for Gram-negative bacterial endotoxin (Cambrex Corporation, 8830 Biggs Ford Road, Walkersville – USA). Gram-negative bacterial endotoxin catalyzes the activation of a proenzyme in the LAL, and the initial rate of activation is directly determined by the concentration of endotoxin. The activated enzyme catalyzes the splitting of p-nitroaniline (pNA) from the colorless substrate Ac-lle-Glu-Ala-Arg-pNA, and the released pNA was measured photometrically at 405–410 nm following termination of the reaction. The correlation between the absorbance and the endotoxin concentration was linear in the range of 0.1–1.0 EU/ml. For the purposes of this study, all samples were run in duplicate within the same plate; therefore, no interassay variability was observed in this study.

To assess recovery of endotoxin within the assay, known concentrations of recombinant endotoxin (0.25 and 1.00 EU/ml) were added to diluted plasma to determine whether the expected concentration correlated with the actual observed value and whether there were any variations due to reaction with plasma contents. Lyophilized endotoxin (E. coli origin) was used to generate a standard curve with the chromogenic LAL test kit in accordance with the manufacturer's instructions.

### Visceral and subcutaneous adiposity measurements

All abdominal ultrasonographic procedures and measurements of visceral and subcutaneous fat tissue were performed by the same physician who was blinded to the subjects’ assignment group. This physician was a specialist in imaging diagnostics using a 3.5-MHz multifrequency transducer (broad band), which reduces the risk of misclassification. The intra-examination coefficient of variation for US was 0.8%.

We took US measurements of intra-abdominal (¨visceral¨) and subcutaneous fat. Ultrasound-determined subcutaneous fat was defined as the distance between the skin and external face of the recto abdominis muscle, and visceral fat was defined as the distance between the internal face of the same muscle and the anterior wall of the aorta. Cutoff points to define visceral obesity by ultrasonographic parameters were based on previous methodological descriptions [25].

### Clinical intervention

#### Dietary program

Energy intake was set at the levels recommended by the dietary reference intake for subjects with low levels of physical activity of the same age and gender following a balanced diet as previously described [20]. No drugs or antioxidants were recommended. Once a week, adolescents had a dietetics lesson, which provided information on the food pyramid, diet record assessment, weight loss diets and miracle diets, food labels, dietetics, fat-free and low-calorie foods, fats (kinds, sources and substitute foods), fast food calories and nutritional composition, good nutritional choices in special occasions, healthy sandwiches, shakes and products to promote weight loss, functional foods and decisions on food choices. All patients received individual nutritional consultation during the intervention program. In addition, a dietitian encouraged the parents to call if they needed extra information.

#### Exercise program

During the one-year interdisciplinary intervention period, adolescents followed a personalized aerobic training program that included a 60-minute session completed three times each week (180-minute/week) under the supervision of a sports therapist. Each program was developed according to the results of an initial oxygen uptake test for aerobic exercises (cycle-ergometer and treadmill). The intensity was set at a work load corresponding to a ventilatory threshold of 1 (50% to 70% of oxygen uptake test). Adolescents were under heart-rate monitoring during the aerobic sessions. The exercise program was based on the 2009 recommendations of the American College of Sports Medicine [26].

#### Psychological intervention

Diagnoses of common psychological problems associated with obesity, such as depression, disturbances of body image, anxiety and decreased self-esteem, were established by validated questionnaires. During the interdisciplinary intervention, the adolescents had weekly psychological support group sessions. During these sessions, the adolescents discussed body image; alimentary disorders, including bulimia, anorexia nervosa and binge eating; the signs, symptoms and health consequences of these disorders; the relationship between feelings and food; and family problems, such as alcoholism. Individual psychological therapy was recommended if individuals were found to have nutritional or behavioral problems [16].

#### Statistical analysis

The data distribution was checked by Bartlett's test for equal variances, and the data are reported as the mean ± SD. Statistical outliers within each treatment group were identified using Grubbs’ test (GraphPad Software) and subsequently removed. All remaining data were analyzed by GraphPad Prism (version 5.00). The differences between groups for all parameters were assessed by a paired *Student’s t test*. The Pearson correlation coefficient was calculated to assess the relationship between variables, and all analyses were carried out with the significance level set at p < 0.05.

## Results

Long-term therapy was effective in reducing body weight (15%), BMI (15%), percent fat (24%), and fat mass (33%). These results are shown in Table 1.

**Table 1:** Effect of long-term multidisciplinary lifestyle therapy on body fat (n = 18)

**Variables**	**Before**	**After**	**% Change**	**p Value**
**Age (yrs)**	15 ± 1.73	16 ± 0.63	7	0.11
**Body weight (kg)**	106.49 ± 11.25	90.53 ± 10.98	−15	<0.001
**Fat mass (kg)**	49.6 ± 10.1	32.9 ± 12.3	−33	<0.001
**Fat-free mass (kg)**	55.8 ± 7.49	58.1 ± 7.71	+4	0.19
**BMI (kg/m**^**2**^**)**	37.98 ± 4.60	32.31 ± 4.44	−15	<0.001
**Percent fat** (%)	47.50 ± 6.99	35.81 ± 9.60	−24	<0.001

Results are expressed as mean value ± SD.

Characteristics of the food intake in obese adolescents are shown in Table 2. Energy intake was reduced 38%, carbohydrate intake was reduced 28%, protein intake was reduced 43%, and fat intake was reduced 47%.

**Table 2:** Characteristics of the food intake in obese adolescents

**Variables**	**Baseline**	**After 1 year**
**Energy intake (kcal)**	1988.04 ± 632.91	1226.77 ± 392.66*
**Carbohydrate (g)**	235.34 ± 92.24	169.54 ± 47.35*
**Carbohydrate (%)**	49.48 ± 7.04	58.39 ± 14.30*
**Protein (g)**	94.42 ± 36.98	53.08 ± 18.58*
**Protein (%)**	18.58 ± 4.33	17.32 ± 3.83
**Lipids (g)**	71.75 ± 29.91	37.72 ± 19.28*
**Lipid (%)**	32.03 ± 4.63	26.20 ± 7.9*

* After 1 year *vs.* Baseline data p ≤ 0.01.

Glucose, insulin, IL-6, adiponectin and endotoxin levels as well as the HOMA-IR are shown in Table 3. Glucose (the range before therapy was 4.38 – 5.66 μU/mL, and the range after therapy was 4.32 – 4.77 μU/mL), insulin (the range before therapy was 7.10 – 23.3 μU/mL, and the range after therapy was 5.00 – 18.9 μU/mL), IL-6 (the range before therapy was 0.97 – 2.22 in Log pg/mL, and the range after therapy was 0.97 – 2.30 Log in pg/mL), adiponectin (the range before therapy was 0.78 – 1.14 Log in ng/mL, and the range after therapy was 0.92 – 1.20 Log in ng/mL), HOMA-IR (the range before therapy was 1.45 – 5.23 μU/mL, and the range after therapy was 1.01 – 3.87 μU/mL), and endotoxin (the range before therapy was 0.112 – 0.394 in Log EU/mL, and the range after therapy was 0.095 – 0.309 in Log EU/mL) were reduced after therapy.

**Table 3:** Glucose, insulin, HOMA index and endotoxin levels in obese adolescents (n = 18)

**Parameters**	**Before**	**After**	**% Change**	**p Value**
**Glucose (μU/mL)**	4.91 ± 0.41	4.55 ± 0.12	−7.2	0.02
**Insulin (ng/mL)**	17.45 ± 4.86	10.21 ± 4.33	- 41	0.001
**HOMA index**	3.82 ± 1.11	2.07 ± 0.91	- 45	0.002
**IL-6 (Log- pg/mL)**	1.63 ± 0.38	1.53 ± 0.33	- 7	0.06
**Endotoxin (Log- EU/mL)**	0.237 ± 0.06	0.188 ± 0.04	- 21	0.0003
**Adiponectin (Log- ng/mL)**	0.979 ± 0.09	1.092 ± 0.07	+12	0.001

Results are expressed as mean value ± SD.

**Figure 1:**
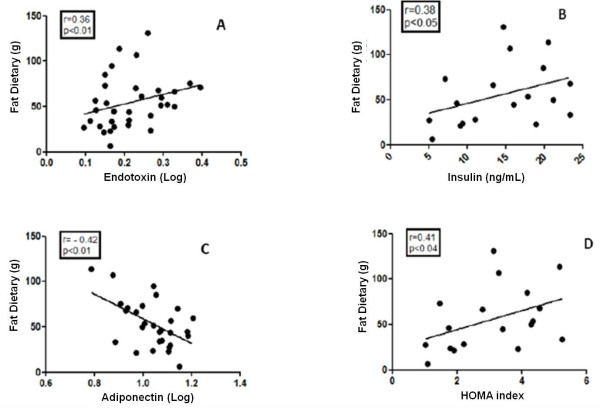
A-D Correlation Pearson between of fat diet intake and endotoxin, insulin, adiponectin and HOMA level in obese adolescents (n = 18).

Pearson correlation analyses showed positive correlations between dietary fat intake and endotoxin levels (r = 0.36, p < 0.01), dietary fat diet intake and insulin levels (r = 0.38, p < 0.05), and dietary fat intake and HOMA-IR (r = 0.41, p < 0.04). In addition, there was a negative correlation between dietary fat intake and adiponectin (r = −0.42, p < 0.01) (Figure 1A-D).

**Figure 2:**
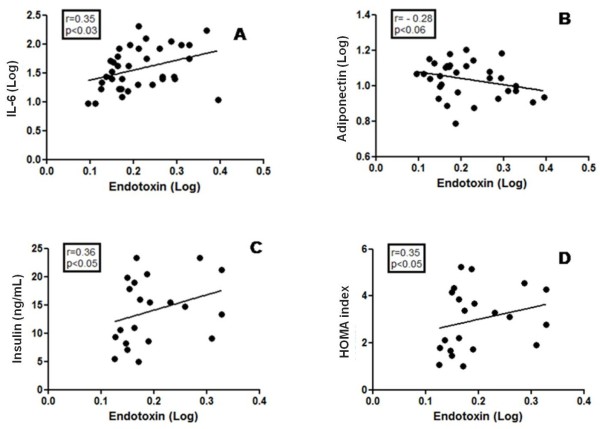
A-D Correlation Pearson between of endotoxin and IL-6, insulin, adiponectin and HOMA level in obese adolescents (n = 18).

We also observed positive correlations between endotoxin and IL-6 levels (r = 0.35, p < 0.03), endotoxin and insulin levels (r = 0.36, p < 0.05), and endotoxin levels and HOMA (r = 0.35, p < 0.05). Interestingly, we observed a negative correlation between endotoxin and adiponectin levels (r = −0.28, p < 0.06) (Figure 2A-D).

## Discussion

The present study showed that long-term therapy was effective in reducing dietary fat intake and endotoxin levels. In addition, these data were positively correlated with improvements in insulin resistance in obese adolescents.

The chronic endotoxemia, have promoted glucose intolerance and hepatic insulin resistance, suggesting its role as a link between innate immunity, inflammation, and insulin resistance [27].

Decreased endotoxin levels have been found with consumption of a low-fat diet compared with a high-fat diet [28-30]. In addition, the type of fatty acid in the diet could have important effects on endotoxinemia. Recently, several studies have shown that omega-3 (ω-3) fatty acids, particularly eicosapentaenoic acid (EPA), reduces endotoxin and pro-inflammatory cytokine concentrations [29,30]. Moreover, Oz et al. [31] demonstrated that a diet rich in EPA, docosahexaenoic acid (DHA), and prebiotic fructooligosaccharides (FOS) protects against LPS-induced systemic inflammatory responses. In contrast to the present study, Al-Attas et al. [3] showed that a diet-controlled program in diabetic individuals did not significantly decrease endotoxin levels compared with individuals who only received insulin. Interestingly, herbs used in food dishes reduce the production of LPS and pro-inflammatory cytokines [32]. In the present study, we observed that interdisciplinary therapy was able to decrease the fat intake, which least in part, was sufficient to reduce endotoxin concentrations and insulin resistance. The reduced endotoxin levels to be related with recovery metabolism and inflammation status that leads to anti-inflammatory status.

The present study found a positive correlation between endotoxin and both pro-inflammatory cytokines (especially, IL-6) and insulin resistance. After interdisciplinary therapy, endotoxinemia, pro-inflammatory status and insulin resistance were decreased. These results showed the importance of making lifestyle changes (i.e., nutritional modification) to reduce the pro-inflammatory state in obese individuals. We have previously shown that long-term therapy is effective in reducing body fat (especially visceral fat), TNF-α and IL-6 and increasing IL-10 and adiponectin. In addition, we observed a positive correlation between pro-inflammatory cytokines (IL-6 and TNF-α levels) and visceral fat [20]. In addition, we can suggested a significant improvement of inflammatory profile once adiponectin, the anti-inflammatory adipokine, increased significantly after the long-term intervention (12%) and reduction of HOMA-IR, an index that present association to inflammatory pathways, such as increase of IL-6 and TNF-alpha.

Creely et al. [5] found that circulating serum endotoxin was higher in type 2 diabetes mellitus (T2DM) patients than in lean healthy subjects, and endotoxin can activate the innate immune pathway in isolated abdominal adipocytes to stimulate secretion of pro-inflammatory cytokines. Creely et al. [5] suggested that the subclinical inflammation seen in type 2 diabetes patients was related to the increase in endotoxin. Mehta et al. [33] observed that endotoxemia (3 ng/kg intravenous bolus in healthy adults) induced an elevation in TNF-α and systemic insulin resistance in humans. Furthermore, insulin resistance measured at 24 h post-LPS was preceded by specific modulation of adipose inflammatory and insulin signaling pathways. Leuwer et al. [34] have shown that endotoxemia leads to major increases in inflammatory adipokine (TNF-α, IL-6, and MCP-1) gene expression in white adipose tissue in mice. In addition, previous studies in human adipose tissue have shown that obesity and T2DM induce upregulation of inflammatory genes [5]. These results are in agreement with the present study in which endotoxin showed a close correlation with IL-6, which was reduced after 1 year of interdisciplinary therapy.

Although we did not directly analyze the effect of exercise, we cannot exclude that the exercise protocol used in the present study contributed to the beneficial effects of the interdisciplinary therapy in obese adolescents. Many studies [11,35-37] have actually demonstrated the benefits of exercise training, which induces an anti-inflammatory state in obese rat and human models. Bradley et al. [36] suggested that voluntary exercise in diet-induced obese mice reduced adiposity despite continued consumption of a high-fat diet. In addition, exercise normalized insulin sensitivity and decreased adipose tissue inflammation (reduced IKK-β gene expression) in these obese mice. These data demonstrated the positive role of exercise training in preventing the development of several diseases, including obesity, diabetes, and fatty liver.

Starkie et al. [38] demonstrated that an intravenous infusion of endotoxin induced a two to threefold increase in the plasma TNF-α level. When human subjects adopted an acute exercise protocol (75% VO_2max_), however, the production of TNF-α elicited by low-grade endotoxemia was inhibited. Similarly, Chen et al. [39] found that chronically exercised rats exhibited minor pathological changes in the heart, liver, and lung after endotoxemia. In addition, Lira et al. [40] observed that a lifestyle change associated with high-intensity, high-volume exercise induced favorable changes in chronic low-grade inflammatory markers and may reduce the risk for obesity, diabetes and cardiovascular diseases.

Although the small number of participants could be a limitation of the present study, the results contribute to the understanding of the mechanisms linking insulin resistance, adiponectinemia in obesity and endotoxemia to the inflammatory state. In addition, the present study highlights the importance of lifestyle interdisciplinary therapy intervention as clinical practice for obesity treatment.

In summary, the present study demonstrated an important association between dietary fat intake and endotoxin level, which reduced significantly after the long-term intervention. Indeed, based on these data, we can hypothesize a link between dietary fat intake, insulin resistance, endotoxin and inflammatory pathways. Taken together, these results suggest that interdisciplinary therapy is effective in decreasing inflammatory markers related to obesity.

## Competing interests

The authors declare that they have no competing interests.

## Authors’ contributions

FSL, JCR, GDP participated of sample collected, assess samples, design of the study and performed the statistical analysis, and writing of paper, RVS, ST and MS helped carry out design of the study, JC, PLS, AP and LT participated of data collection and analysis, MTM, CMON, LMO, CTS and ARD performed the design of the study and discussion of paper. All authors read and approved the final manuscript and apply to add the new author.
